# Identification and validation of a novel Parkinson-Glioma feature gene signature in glioma and Parkinson’s disease

**DOI:** 10.3389/fnagi.2024.1352681

**Published:** 2024-05-30

**Authors:** Hengrui Zhang, Jiwei Wang, Nan Su, Ning Yang, Xinyu Wang, Chao Li

**Affiliations:** ^1^Department of Neurosurgery, Qilu Hospital, Cheeloo College of Medicine and Institute of Brain and Brain-Inspired Science, Shandong University, Jinan, China; ^2^Jinan Microecological Biomedicine Shandong Laboratory and Shandong Key Laboratory of Brain Function Remodeling, Jinan, China

**Keywords:** Parkinson’s disease, glioma, machine learning algorithms, gene signature, prognosis

## Abstract

**Introduction:**

The prognosis for glioma is generally poor, and the 5-year survival rate for patients with this disease has not shown significant improvement over the past few decades. Parkinson’s disease (PD) is a prevalent movement disorder, ranking as the second most common neurodegenerative disease after Alzheimer’s disease. Although Parkinson’s disease and glioma are distinct diseases, they may share certain underlying biological pathways that contribute to their development.

**Objective:**

This study aims to investigate the involvement of genes associated with Parkinson’s disease in the development and prognosis of glioma.

**Methods:**

We obtained datasets from the TCGA, CGGA, and GEO databases, which included RNA sequencing data and clinical information of glioma and Parkinson’s patients. Eight machine learning algorithms were used to identify Parkinson-Glioma feature genes (PGFGs). PGFGs associated with glioma prognosis were identified through univariate Cox analysis. A risk signature was constructed based on PGFGs using Cox regression analysis and the Least Absolute Shrinkage and Selection Operator (LASSO) method. We subsequently validated its predictive ability using various methods, including ROC curves, calibration curves, KM survival analysis, C-index, DCA, independent prognostic analysis, and stratified analysis. To validate the reproducibility of the results, similar work was performed on three external test datasets. Additionally, a meta-analysis was employed to observe the heterogeneity and consistency of the signature across different datasets. We also compared the differences in genomic variations, functional enrichment, immune infiltration, and drug sensitivity analysis based on risk scores. This exploration aimed to uncover potential mechanisms of glioma occurrence and prognosis.

**Results:**

We identified 30 PGFGs, of which 25 were found to be significantly associated with glioma survival. The prognostic signature, consisting of 19 genes, demonstrated excellent predictive performance for 1-, 2-, and 3-year overall survival (OS) of glioma. The signature emerged as an independent prognostic factor for glioma overall survival (OS), surpassing the predictive performance of traditional clinical variables. Notably, we observed differences in the tumor microenvironment (TME), levels of immune cell infiltration, immune gene expression, and drug resistance analysis among distinct risk groups. These findings may have significant implications for the clinical treatment of glioma patients.

**Conclusion:**

The expression of genes related to Parkinson’s disease is closely associated with the immune status and prognosis of glioma patients, potentially regulating glioma pathogenesis through multiple mechanisms. The interaction between genes associated with Parkinson’s disease and the immune system during glioma development provides novel insights into the molecular mechanisms and targeted therapies for glioma.

## 1 Introduction

Glioma, the most common malignant tumor of the nervous system, is associated with a poor prognosis and treatment challenges. Glioma is commonly regarded as one of the most formidable tumors specific to the central nervous system (CNS), characterized by the rapid proliferation of cancerous glial cells. It accounts for nearly half of all brain tumors, with an annual incidence rate ranging from 30 to 80 cases per million population (Li et al., 2022). Glioma can be caused by various environmental factors, including high-dose radiation, petroleum, and vinyl chloride. Genetic factors also play a significant role in the onset of glioma. Additionally, viral infections, head trauma, immune suppression, and endocrine disorders are also linked to the occurrence and development of gliomas (Preusser et al., 2006).

Currently, traditional treatments for gliomas primarily consist of surgery, chemotherapy, and radiation therapy. The standard treatment for gliomas is maximal surgical resection combined with radiotherapy and chemotherapy (Stupp et al., 2005). However, the efficacy of these traditional methods is limited, as they can only provide partial relief for glioma symptoms, and there is a risk of adverse drug resistance, recurrence, and metastasis (de Blank et al., 2020). Despite significant advancements in glioma treatment in recent years, the therapeutic outcomes still fall short of expectations, and the prognosis remains poor. Therefore, it is crucial to investigate the molecular mechanisms underlying the development and progression of gliomas, aiming to identify novel prognostic biomarkers and therapeutic targets.

Parkinson’s disease (PD) is a prevalent movement disorder and the second most common neurodegenerative disease, following Alzheimer’s disease (Hickman et al., 2018). Most cases of Parkinson’s disease are idiopathic, with the incidence rate gradually increasing with age. Additionally, Parkinson’s disease is influenced by factors such as genetics, environment, and oxidative stress (Fung et al., 2017). The risk of Parkinson’s disease may increase due to exposure to toxic chemicals like pesticides and herbicides, as well as head injuries, while certain lifestyle factors like smoking and caffeine intake may decrease the risk (Ritz et al., 2007; Kenborg et al., 2015; Simon et al., 2020). The diagnosis of Parkinson’s disease primarily relies on the typical clinical manifestations observed in the late stages of the disease. Parkinson’s disease is characterized by motor symptoms (e.g., bradykinesia, rigidity, and tremors) and non-motor features (e.g., constipation, urinary frequency, functional decline, depression, cognitive impairment, and sleep disorders), which significantly impact the patients’ quality of life (Armstrong and Okun, 2020; Vijiaratnam et al., 2021).

Treatment measures for Parkinson’s disease include medication, rehabilitation therapy, exercise, palliative care, and surgery (Armstrong and Okun, 2020). However, thus far, no measures have been definitively proven to delay or halt the progression of the disease. Therefore, the pursuit of finding interventions that can delay or prevent the progression of Parkinson’s disease is a major goal for both researchers and patients (Armstrong and Okun, 2020).

Cancer is a complex disease characterized by uncontrolled cell proliferation and metastasis (Siegel et al., 2015). Cancer and Parkinson’s disease can be considered as opposing conditions in terms of pathogenesis, with cancer arising from uncontrolled cell division and Parkinson’s disease from cell death. Numerous studies confirm that patients with PD have a lower risk of cancer, especially central nervous system tumors (Diamandis et al., 2009; Becker et al., 2010; Park et al., 2019). However, a meta-analysis involving 40 studies, 2,317,408 cases, and 12,113,484 control subjects indicated that patients with PD were significantly associated with a reduced risk of lung cancer, genitourinary cancers, gastrointestinal cancers and hematological cancers, but a higher occurrence of melanoma and brain cancer (Leong et al., 2021). Therefore, exploring PD-related genes in the diagnosis of gliomas holds great promise and may also provide value in the diagnosis and prognosis of gliomas.

In this study, we developed a signature using Parkinson-Glioma feature genes (PGFGs). Through systematic analysis, the 19-gene signature demonstrated good accuracy in predicting the survival time of glioma patients and functioned as an independent prognostic factor for glioma. Furthermore, its involvement in reshaping the tumor microenvironment offers new insights into the molecular mechanisms and targeted treatment of glioma. The workflow diagram of this study is presented in Supplementary Figure 1.

## 2 Materials and methods

### 2.1 Data retrieval and preprocessing

Firstly, we downloaded HTSeq-FPKM gene expression data and related clinical information of glioma patients from The Cancer Genome Atlas (TCGA) database as the training set. Our sample exclusion criteria were as follows: Firstly, patients with incomplete survival data were excluded. This included those with unclear survival time (i.e., null value) or ambiguous survival status (i.e., null value). Secondly, patients with a follow-up time of less than 30 days were excluded. We were concerned that a short follow-up duration may introduce bias; therefore, these patients were filtered out. If a patient dies within a short period of time, the cause of death is highly likely to involve non-tumor-related factors, making it of limited value for predicting long-term prognosis. After excluding patients who met the exclusion criteria, a total of 631 glioma patients were enrolled. In the process of further validation, we applied the same exclusion criteria.

A total of 618 glioma patients in dataset CGGA-693 and 306 glioma patients in dataset CGGA-325 were obtained from the China Glioma Genome Atlas (CGGA) data portal. A total of 249 glioma patients were included in the GSE16011 dataset obtained from the NCBI Gene Expression Omnibus (GEO).^1^ The “Combat” algorithm of the R package “sva” was used to reduce the possibility of batch effect due to non-biological bias between the TCGA-Glioma dataset and the CGGA-693, CGGA-325 and GSE16011 datasets (Johnson et al., 2007). In addition, three PD datasets, GSE49036, GSE20141 and GSE7621, were obtained from the GEO cohort to identify PD related genes. The GSE7621 dataset included 16 brain samples from PD patients and 9 normal brain samples from controls. The GSE20141 dataset included 10 brain samples from PD patients and 8 normal brain samples from controls. The GSE 49036 dataset included 20 brain samples from PD patients and 8 normal brain samples from controls. The three PD datasets were generated using the GPL570 (HG-U133 Plus 2) Affymetrix Human Genome U133 Plus 2.0 array. Similarly, we used the “Combat” algorithm to remove the non-biological effects.

### 2.2 Eight machine learning algorithms for identifying Parkinson-Glioma feature genes

We utilized the “limma” package in R to detect genes that were differentially expressed between Parkinson’s disease (PD) brain tissue and normal brain tissue. To increase the sample size of normal tissue, we integrated the glioma expression data with the normal brain tissue data from GTEx (Genotype-Tissue Expression). This allowed us to identify differentially expressed genes that may have predictive value for glioma diagnosis.

Based on the “caret” package in R, we used the genes obtained above to construct a characteristic diagnostic model, including random forest (RF) model, support vector machine (SVM) learning model, extreme gradient boosting (XGBoost) model, generalized linear model (GLM), elastic net model, stepLDA (Linear Discriminant Analysis with Stepwise Feature Selection) model, Partial Least Squares (PLS) model and Multi-Step Adaptive MCP-Net (msaenet) model. Following the modeling with the aforementioned eight methods, we performed residual analysis on the data samples. Subsequently, we generated reverse cumulative distribution plots for the residuals of each method, allowing us to evaluate the machine learning accuracy based on the reverse cumulative residual scores. Parkinson-Glioma feature gene (PGFG) was identified according to Root Mean Square Error (RMSE).

### 2.3 Gene signature construction and evaluation

Univariate Cox analysis was performed on the TCGA-Glioma, CGGA-693, CGGA-325, and GSE16011 datasets using the “survival” package in R. To ensure result reliability, we used a stringent threshold of *p* < 0.001 to identify PGFGs associated with prognosis. The TCGA glioma patient cohort was utilized as the training set. Lasso-Cox regression analysis was employed to screen and eliminate collinearity among 25 prognosis-related PGFGs. Subsequently, a risk score model was constructed by multiplying the β (Coef) value with the PGFG expression levels. Risk score = (β1*PGFG1 + β2* PGFG2 + β3* PGFG3 + ⋯ + βn* PGFGn), where β represents the coefficient of the PGFG (Friedman et al., 2010; Simon et al., 2011).

We calculated the risk scores of patients in the CGGA-693, CGGA-325, and GSE16011 datasets. Then, we conducted univariate Cox analysis on the risk scores and performed a meta-analysis using the “meta” package to assess the consistency and heterogeneity of the prognostic model across all four datasets. We used the median risk score in the training set as the cutoff value to classify patients in the four datasets into high-risk and low-risk groups. Subsequently, we performed Kaplan–Meier survival analysis, ROC curve analysis, and calibration curve analysis to evaluate the prognostic model’s predictive ability, accuracy, and repeatability in glioma patients. In the training set, we performed univariate and multivariate Cox analysis on risk scores, Age, Gender, and Grade staging to determine the independence of the signature from traditional clinical variables. We employed the concordance index method to assess the accuracy advantage of the signature, and decision curve analysis (DCA) to evaluate its potential benefits for patients.

### 2.4 Exploring the potential mechanisms of glioma

We utilized eight software tools [MCPcounter (Becht et al., 2016), CIBERSORT (Newman et al., 2015), xCell (Aran et al., 2017), TIMER (Li et al., 2017), EPIC (van Veldhoven et al., 2011), QUANTISEQ (Finotello et al., 2019), estimate (Yoshihara et al., 2013), IPS (Charoentong et al., 2017)] to quantify the abundance of immune infiltration in patients. Next, we compared the differences in immune infiltration between high-risk and low-risk groups and calculated the Pearson correlation between the genes, signatures (risk scores) in the model, and the content of immune cells. The signature effectively quantified the risk of glioma patients. To explore the significant heterogeneity between the high-risk group and the low-risk group, we constructed a weighted gene co-expression network using the “WGCNA” R package, known for its approximate scale-free characteristics (Langfelder and Horvath, 2012). Highly coordinated genes were identified based on the correlation among all these gene expression values. The network module was generated using the Topological Overlap Measure (TOM; Li and Horvath, 2009), and co-expressed gene modules were identified using the Dynamic Hybrid Cutting method (a bottom-up algorithm; Langfelder et al., 2008). Finally, modules with related genes were merged.

The correlation between genes and modules was measured by calculating gene significance (GS) and module significance (MS). Furthermore, we identified significantly co-expressed gene modules in the high-risk group using the WGCNA algorithm and conducted functional enrichment analysis on these modules.

Additionally, we compared the differences in four groups of immune-related genes (Immunoinhibitor, Chemokines, Immunostimulator, Human Leukocyte Antigen) between the high-risk and low-risk groups.

We conducted GSEA enrichment analysis on the C2, C5, and Hallmark gene sets in the MSigDB database and the CancerSEA database, which defines 14 tumor states. We used the “clusterProfiler” package to thoroughly analyze the differences in pathway activation between the high-risk and low-risk groups (Yu et al., 2012; Wu et al., 2021). By using butterfly diagrams, we vividly displayed the correlation between risk scores and TIP (Tracking Tumor Immunophenotype) scores, 8 types of immunotherapy scores, and different tumor signaling pathways.

### 2.5 Applying a signature to guide chemotherapy and immunotherapy

Chemotherapy is a commonly used treatment for patients with glioma. To predict the chemotherapy response, we utilized the R package “pRRophetic” to estimate the half-maximal inhibitory concentration (IC50) of chemotherapeutic drugs in different patient subtypes (Geeleher et al., 2014). Immunotherapy represents a novel treatment approach. To assess the predictive performance of our model for immunotherapy, we compared the scores of eight different types of immunotherapies between high-risk and low-risk groups.

## 3 Results

### 3.1 Identification of 30 Parkinson-Glioma feature genes

**Figure 1:**
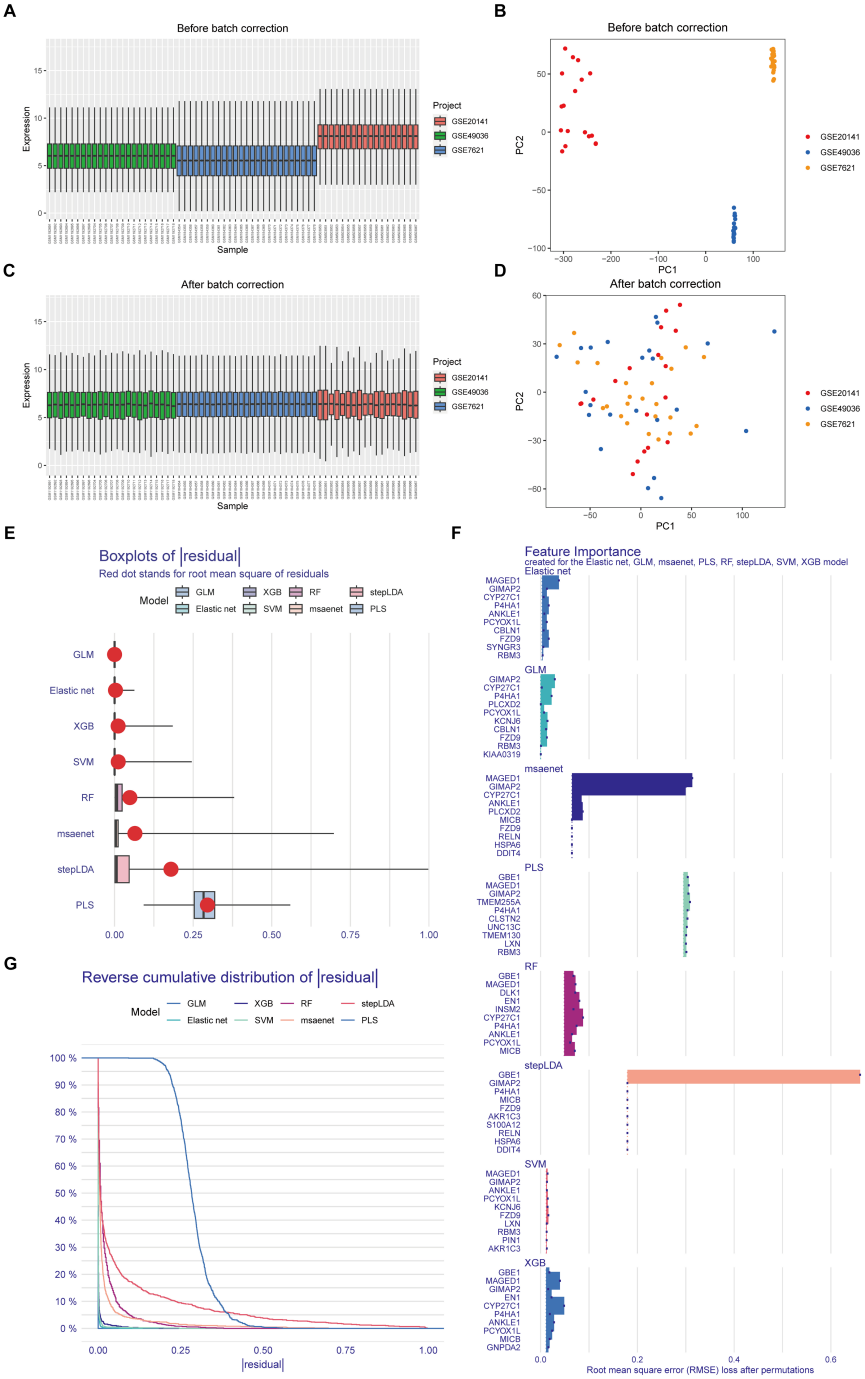
Identification of Parkinson-Glioma feature genes. GSE7621: 9 normal brain samples and 16 PD brain samples GSE20141: 8 normal brain samples and 10 PD brain samples GSE49036: 8 normal brain samples and 20 PD brain samples. **(A)** The boxplot illustrated the distribution of gene expression before the removal of batch effects; **(B)** Principal Component Analysis (PCA) demonstrated expression patterns before the elimination of batch effects; **(C)** The boxplot depicted the distribution of gene expression after batch effect removal; **(D)** PCA displayed expression patterns following batch effect removal; **(E)** Box plots of sample residuals from the eight algorithms were presented. The x-axis represented the quantile of outliers, with the red dot indicating the mean; **(F)** Eight different algorithms identified the top 10 significant genes, resulting in the discovery of 30 Parkinson-Glioma feature genes; **(G)** Reverse Cumulative Distribution Maps of model residuals were constructed using RF, SVM, XGB, GLM, Elastic Net, stepLDA, PLS, and msaenet. The y-axis represented the outlier percentile.

Prior to applying the combat algorithm from the “sva” package for correction, the box plot and principal component analysis revealed notable batch effects in GSE49036, GSE20141, and GSE7621 datasets (Figures 1A,B). We successfully mitigated non-biological biases among the Parkinson’s datasets (Figures 1C,D). Through differential analysis, we identified a total of 112 genes with differential expression. These genes can be found in Supplementary Table 1. Subsequently, we employed eight machine learning algorithms to train diagnostic genes capable of distinguishing gliomas from normal tissues, thus identifying Parkinson-Glioma feature genes. The sum of errors between the residual predicted values and the actual values reflects the prediction accuracy of the model. The residual boxplot (Figure 1E) and the residual reverse cumulative distribution plot (Figure 1G) indicate a high predictive ability of the model. Performance verification of different diagnostic models based on eight algorithms is presented in Supplementary Figure 2. Due to the good accuracy of each algorithm, we determined the top 10 important genes of each algorithm as Parkinson-Glioma feature genes based on RMSE (Figure 1F). After removing duplicate genes, we obtained a total of 30 Parkinson-Glioma feature genes. These genes can be found in Supplementary Table 2.

### 3.2 The 19-PGFGs signature is an independent prognostic factor for gliomas

**Figure 2:**
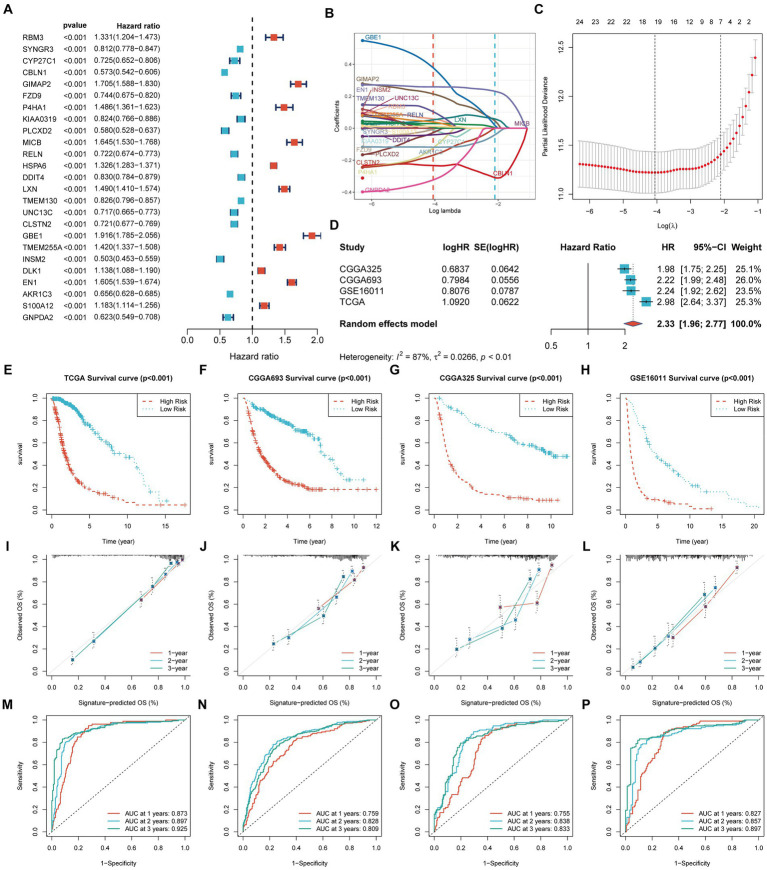
Construction and validation of the prognostic signature. TCGA-Glioma: 631 glioma patients, 315 cases in the high-risk group and 316 cases in the low-risk group CGGA-693: 618 glioma patients, 365 cases in the high-risk group and 253 cases in the low-risk group CGGA-325: 306 glioma patients, 178 cases in the high-risk group and 128 cases in the low-risk group GSE16011: 249 glioma patients, 171 cases in the high-risk group and 78 cases in the low-risk group The significance of the survival curve was evaluated using the log-rank test. **(A)** Univariate Cox analysis identified 25 prognostic genes; **(B,C)** Coefficient profiles of the 19 prognostic PGFGs obtained through Lasso-Cox regression analysis. The Lasso regression model revealed the partial likelihood deviance of variables. Red dots represented the partial likelihood of deviance values, and gray lines represented the standard error (SE). The two vertical dotted lines on the left and right symbolized optimal values based on minimum criteria and 1 − SE criteria, respectively; **(D)** Meta-analysis demonstrated the heterogeneity and consistency of the signature across TCGA-Glioma, CGGA-693, CGGA-325, and GSE16011 datasets; **(E–H)** KM curves illustrated the differences in OS between the high-risk and low-risk groups in the TCGA-Glioma, CGGA-693, CGGA-325, and GSE16011 datasets; **(I–L)** Calibration curves denoted the accuracy and specificity of the signature. ROC curves displayed the 1-, 2-, and 3-year OS in the TCGA-Glioma (**M**), CGGA-693 **(N)**, CGGA-325 **(O)**, and GSE16011 **(P)** datasets.

We identified 25 genes associated with OS in glioma through univariate Cox analysis (Figure 2A). The Lasso-Cox analysis ultimately identified 19 genes for constructing a prognostic model (Figures 2B,C). The risk score = 0.0583 × RBM3 + (−0.0126) × CYP27C1 + (−0.2337) × CBLN1 + 0.2144 × GIMAP2 + (−0.0067) × FZD9 + (−0.1237)× P4HA1+ (−0.0281) × PLCXD2 + 0.0292 × HSPA6 + (−0.0415)× DDIT4 + (0.0521) × LXN + 0.1032 × TMEM130 + (−0.1693) × CLSTN2+ 0.3785 × GBE1 + 0.0388 × TMEM255A + 0.0050 × DLK1 + 0.2588 ×EN1 + (−0.1183) × AKR1C3 + 0.0113 × S100A12 + (−0.3239) × GNPDA2, where β is the coefficient of the PGFG. Nineteen prognostic PGFGs obtained through Lasso-Cox regression analysis and their coefficients can be found in Supplementary Table 3.

**Figure 3:**
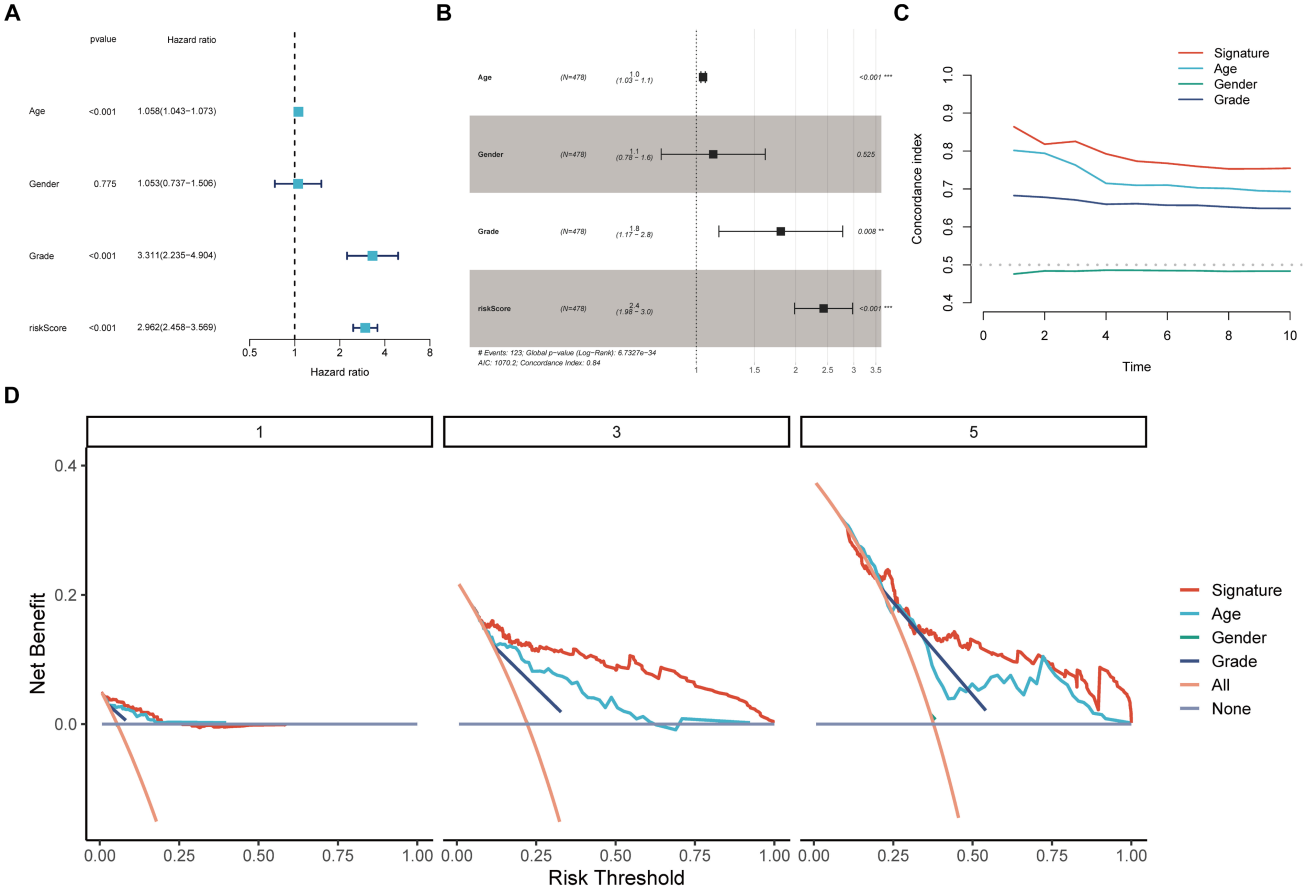
Internal validation of the signature. The univariate **(A)** and multivariate **(B)** Cox regression analyses demonstrated the independent prognostic value of the signature for Glioma patients (*p* < 0.001); **(C)** The time-dependent C-index indicated the accuracy of the signature; **(D)** The DCA curves emphasized the potential clinical benefits of the signature for Glioma patients.

Figure 2D presents the meta-analysis results of the univariate Cox analysis for the prognostic model in four datasets. Despite the heterogeneity among different datasets, these results consistently demonstrate that the 19-PGFGs signature is a significant risk factor for glioma in all four datasets. The median risk score was used as the cut-off value to divide the 631 patients into high-risk and low-risk groups. Similar results were obtained using the same method on the CGGA-693 dataset, the CGGA-325 dataset, and the GSE16011 dataset. The risk scores of patients in four datasets can be found in Supplementary Tables 4–7. The Kaplan–Meier curve illustrates that the OS of the low-risk group is significantly better than that of the high-risk group (Figures 2E–H). The calibration curve analysis indicates a good consistency between the predicted values and the actual values (Figures 2I–L). The model-predicted 1-, 2-, and 3-year OS AUCs validate the predictive performance of the signature, demonstrating satisfactory specificity and sensitivity (Figures 2M–P). Based on the clinical information from TCGA-Glioma, both univariate Cox (Figure 3A) and multivariate Cox (Figure 3B) analyses indicate that the 19-PGFGs signature is an independent prognostic factor for glioma patients (*p* < 0.001). The C-index (Figure 3C) indicates that our signature outperforms traditional clinical variables. DCA (Figure 3D) suggests that applying our model can benefit patients. Our signature demonstrates robustness.

### 3.3 The high-risk group exhibits higher levels of immune cell infiltration

**Figure 4:**
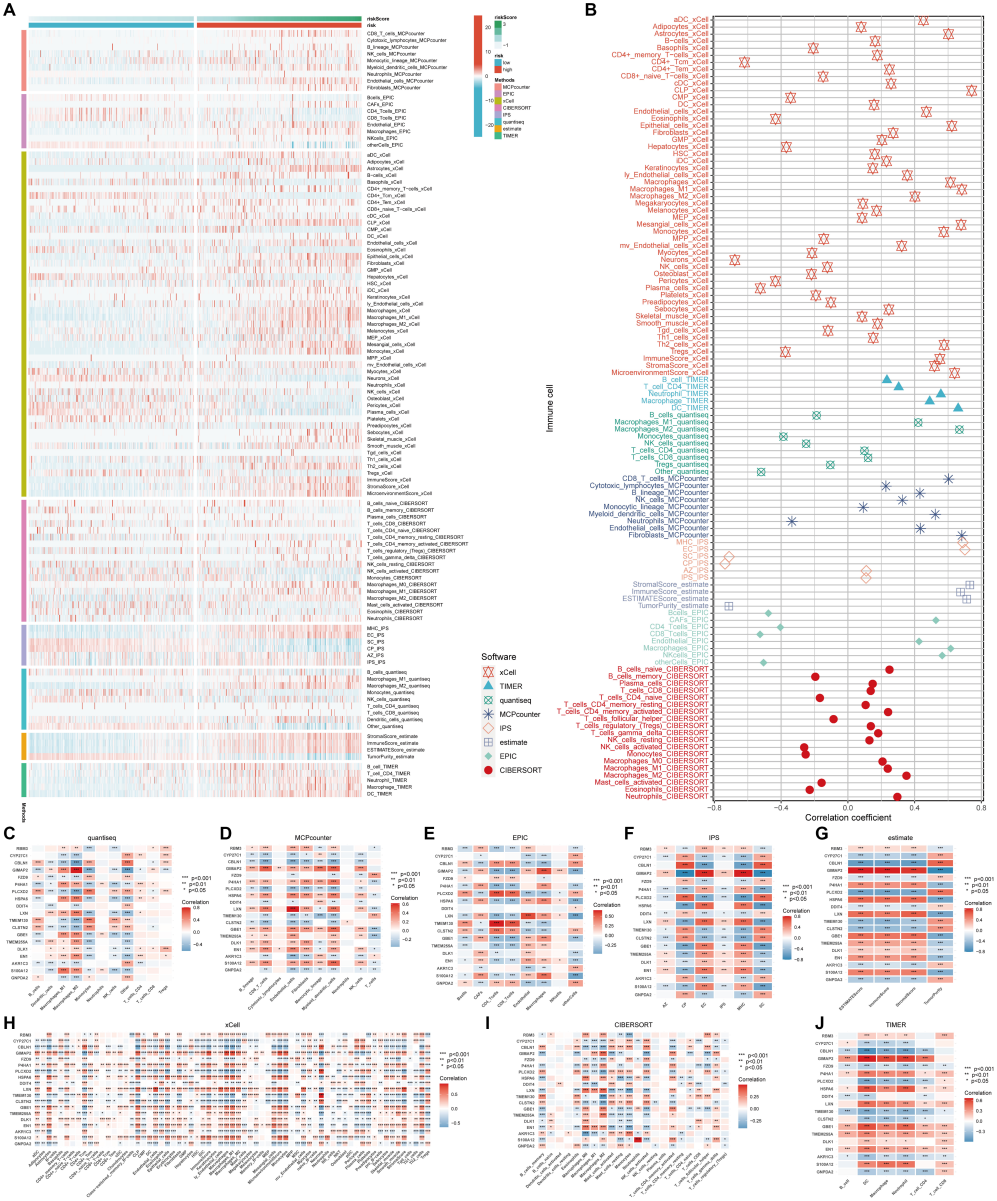
Immune infiltration profiles. The main methods involved were Wilcoxon rank-sum test for difference analysis and Spearman correlation analysis. **(A)** Eight immune infiltration software tools showed varying quantities of immune cells between the high-risk and low-risk groups; **(B)** Correlation between immune cells and risk scores; **(C–J)** Correlation between immune cells and genes incorporated in the model.

To investigate the correlation between risk score and immune cell content, eight different software programs were utilized to evaluate the immune cell content. Subsequently, the Wilcoxon rank sum test was conducted to compare the immune cell content between high and low scoring groups. The results are presented in Figure 4A. Clearly, the high-risk group exhibits a higher number of immune cells. Furthermore, the Spearman’s correlation analysis aligns with the difference analysis, demonstrating a significant positive correlation between the risk score and the content of different immune cell types (Figure 4B).

These findings suggest that this signature may serve as a crucial immune marker. The genes in the model exhibit intricate correlation patterns with immune cell infiltration, suggesting that these genes may be involved in the reshaping of the diversified immune microenvironment (Figures 4C–J).

### 3.4 The high-risk group and the low-risk group exhibit distinct biological patterns

**Figure 5:**
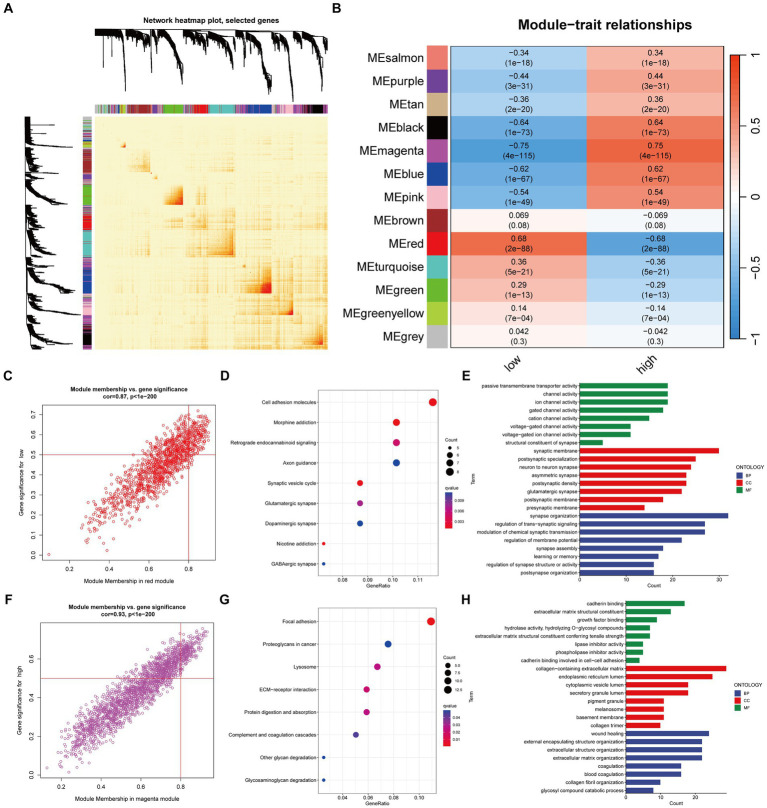
Mining of network modules using WGCNA. The main method involved was Pearson correlation analysis. **(A)** Heatmap of the network for all genes; **(B)** Heatmap showing the correlation between module eigengenes and risk traits; **(C)** Correlation and significance of the red module with the low-risk group of Glioma patients; **(D,E)** GO and KEGG analysis of genes in the red module; **(F)** Correlation and significance of the magenta module with the high-risk group of Glioma patients; **(G,H)** GO and KEGG analysis of genes in the magenta module.

We employed WGCNA (Weighted Gene Co-expression Network Analysis) to correlate gene expression profiles with risk groups and generate heatmaps for visualizing the gene networks. The heatmap illustrated the topological overlap matrix (TOM) among all analyzed genes, ultimately constructing a co-expression atlas (Figure 5A). The high-risk group and the low-risk group exhibit distinct biological patterns due to variations in their co-expression gene modules (Figure 5B).

Specifically, the red module exhibits a significant positive correlation with the low-risk group, while the high-risk group shows a significant positive correlation with the magenta module. The core genes in the module are defined as those with GS > 0.5 and MM > 0.8 (Figures 5C,F). GO and KEGG enrichment analysis suggest that the red module (Figures 5D,E) is enriched in molecular functions such as channel activity, and the magenta module (Figures 5G,H) is enriched in molecular functions such as growth factors.

### 3.5 Molecular expression and pathway activity differ between the high-risk and low-risk groups

**Figure 6:**
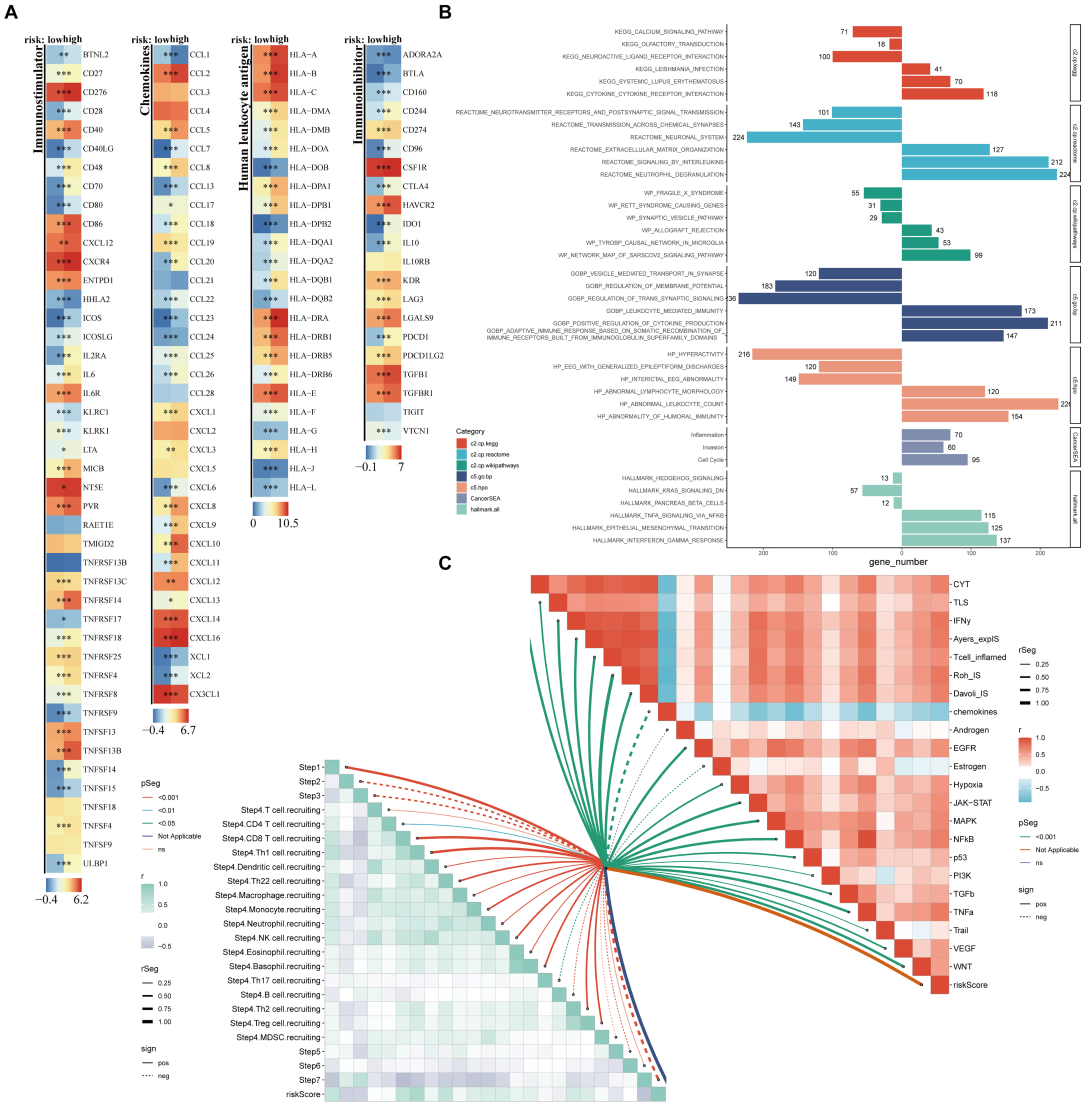
Exploration of potential risk mechanisms. The main methods involved were Wilcoxon rank-sum test for difference analysis, GSEA for enrichment analysis, and Spearman correlation analysis. **(A)** Comparative analysis of four distinct categories of immune-related genes, namely Immunoinhibitor genes, Chemokines, Immunostimulator genes, and Human Leukocyte Antigen, between high- and low-risk groups; **(B)** Bar charts represented the GSEA results; **(C)** Butterfly plots vividly illustrated the correlation between risk score, TIP score, eight immunotherapy scores, and various tumor signaling pathways.

The high-risk group shows high expression of four types of immune-related genes, as indicated by the results of immune infiltration analysis (Figure 6A). GSEA analysis reveals significant activation of malignant tumor characteristics, including inflammatory response, cell cycle, and invasion, in patients belonging to the high-risk group (Figure 6B). Despite exhibiting higher immune cell infiltration, the high-risk group contributes to the development of inflammatory characteristics in glioma patients, thereby increasing malignancy, as supported by previous findings on immune infiltration. This point is further confirmed by the correlation between risk score and TIP score, eight immunotherapy scores, and multiple tumor signaling pathways (Figure 6C).

### 3.6 Chemotherapy and immunotherapy

**Figure 7:**
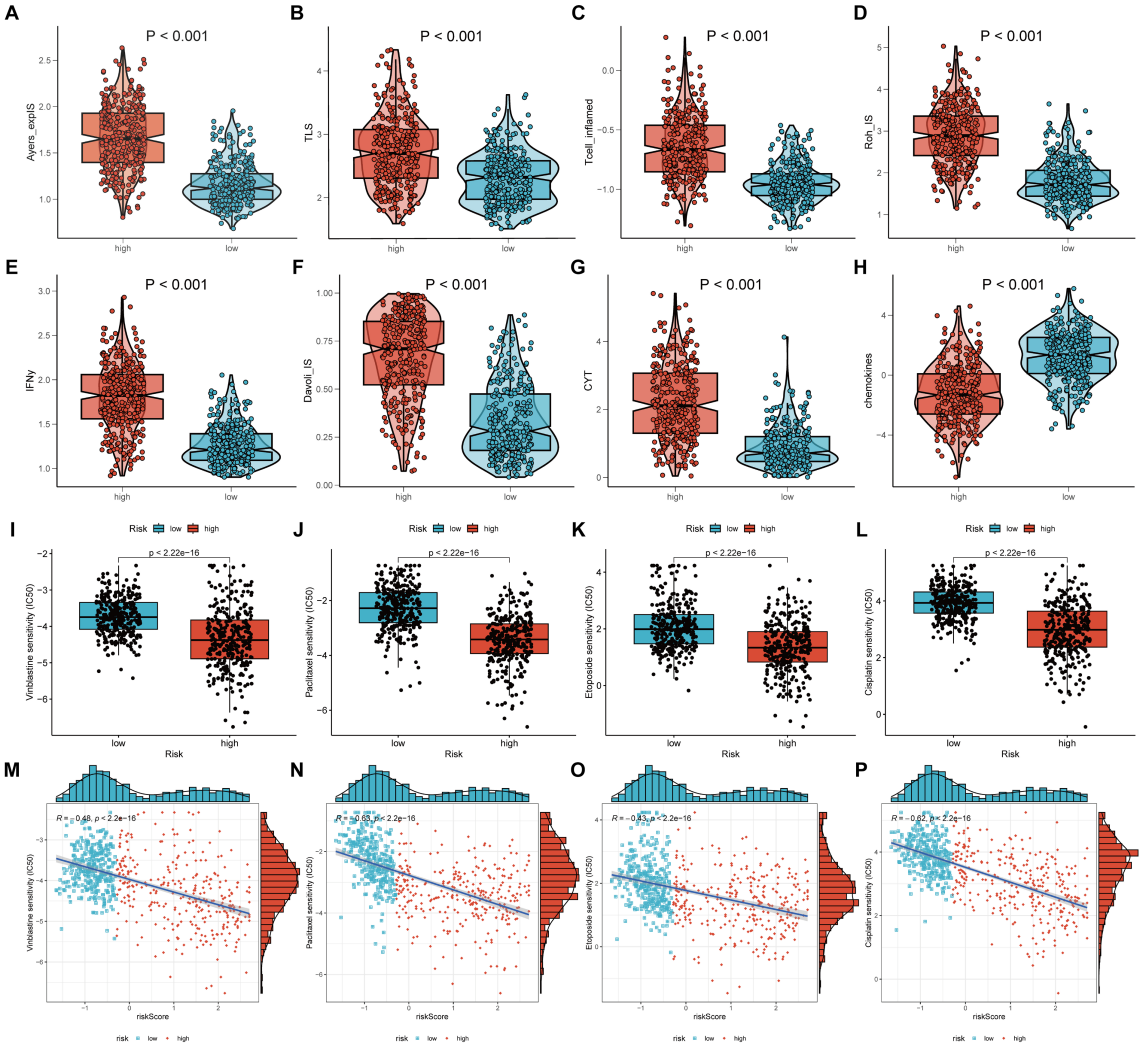
Chemotherapy and immunotherapy. The main methods involved were Wilcoxon rank-sum test for difference analysis and Spearman correlation analysis. **(A–H)** The scores of eight different types of immunotherapies between high-risk and low-risk groups; **(I–L)** The IC50 of four chemotherapy drugs between high-risk and low-risk groups; **(M–P)** The IC50 values of the four chemotherapy drugs showed a significant negative correlation with the risk score.

In addition to the chemokine scoring, the other immunotherapy scores were consistent, suggesting that the high-risk group may be more appropriate for immunotherapy, potentially because they have greater immune reserves (Figures 7A–H). Furthermore, the IC50 values of four chemotherapy drugs were higher in the low-risk group (Figures 7I–L) and showed a significant negative correlation with the risk score (Figures 7M–P). This suggests that patients in the high-risk group may exhibit greater sensitivity to these four drugs.

## 4 Discussion

Gliomas are classified into four categories by the World Health Organization (WHO), with the first two types being low-grade gliomas (LGG) and the last two being high-grade gliomas (HGG; Tom et al., 2019). The poor prognosis of glioma, regardless of its subtype, is attributed to its high heterogeneity, invasiveness, permeability of the blood–brain barrier (BBB), hypoxic tumor niche, and the presence of epithelial-mesenchymal transition (EMT; Śledzińska et al., 2021; Li et al., 2022). Glioblastoma (GBM) has a median survival period of no more than 16 months (Stupp et al., 2009). Current methods for treating gliomas are imprecise and have limited effectiveness (Dono et al., 2021). Accurate prediction of glioma patients’ prognosis is significantly important in guiding their treatment. The microenvironment of glioma involves crosstalk among multiple signaling pathways and biological mechanisms, contributing to its continuous growth and development (Zhao et al., 2021).

Interestingly, the discovered relationship between Parkinson’s disease (PD) and brain cancer suggests that these two distinct diseases, PD and glioma, may share common biological pathways that contribute to their development (Leong et al., 2021). Therefore, developing a prognostic model based on Parkinson’s disease-related genes could be a valuable approach for predicting glioma prognosis.

We analyzed the expression data of 112 genes related to Parkinson’s disease in glioma patients. The data was obtained from the TCGA, CGGA, and GEO databases, and the patients had a follow-up time of more than 30 days. We identified 25 PGFGs that were prognostically significant. The majority of the included PGFGs showed a significant association with overall survival in glioma patients. This finding highlights the important role of PGFGs in glioma and underscores the accuracy of machine learning.

We developed a 19-gene signature that accurately predicts overall survival in glioma patients. The risk score of this signature indicates its potential as an independent prognostic model. We observed a positive correlation between the expression levels of RBM3, GIMAP2, HSPA6, LXN, TMEM130, GBE1, TMEM255A, DLK1, EN1, and S100A12 and the overall survival of gliomas. Conversely, the expression levels of CYP27C1, CBLN1, FZD9, P4HA1, PLCXD2, DDIT4, CLSTN2, AKR1C3, and GNPDA2 showed a negative correlation with the overall survival of gliomas. Previous studies have demonstrated that the majority of the 19 genes comprising prognostic features are associated with cancer or disease progression. They exhibit either favorable or unfavorable prognostic significance in different tumors. This indirectly indicates the biological significance of the selected survival-related genes and the effectiveness of our signature.

RNA-binding motif protein 3 (RBM3) is an excellent cold-shock protein that can rapidly upregulate its expression to ensure homeostasis and survival under cold stress conditions in the body (Ferry et al., 2011; Hu et al., 2022). RBM3 has been confirmed as a neuroprotective protein and demonstrates protective effects in cases of acute brain and spinal cord injuries (Choi et al., 2012). Additionally, RBM3 is closely linked to the development and progression of neurodegenerative diseases, including Lewy body dementia and Alzheimer’s disease (Rajkumar et al., 2020; Hu et al., 2022). Numerous immune studies have demonstrated the upregulation of RBM3 in various tumors, including malignant astrocytoma, classifying it as an oncogene (Zhang et al., 2013; Karnevi et al., 2018; Hu et al., 2022). Interestingly, some studies have indicated a close association between RBM3 overexpression and favorable clinical outcomes, making it a potential biomarker for cancer treatment (Al-Astal et al., 2016). RBM3 exhibits varying effects on prognosis in different cancers, highlighting its involvement in multiple complex mechanisms that necessitate further exploration. Our research results are consistent with previous studies, confirming that RBM3 is an important biomarker positively correlated with glioma prognosis, and also a differentially expressed gene in Parkinson’s disease. It is a gene that is related to both neurodegenerative diseases and cancer. However, the specific mechanism of RBM3 in glioma and Parkinson’s disease is still unclear, and there is a lack of direct evidence. Related research is very limited. Future studies can further explore the expression patterns, regulatory mechanisms of RBM3 in Parkinson’s disease and glioma, as well as its correlation with disease progression, providing new ideas and methods for the diagnosis and treatment of these two diseases.

Glycogen branching enzyme (GBE1) is a critical gene that participates in regulating glycogen metabolism. Additionally, GBE1 exhibits varying effects on the prognosis of different cancers (Lando et al., 2009; Liang et al., 2021). GBE1 has been shown to impact FBP1 expression via the NF-κB pathway, thereby influencing the glucose metabolism pattern of glioma cells (Chen et al., 2023). This promotes tumor progression driven by the Warburg effect, which is associated with a poor prognosis in glioma (Chen et al., 2023). Our research indicates that there is a positive correlation between GBE1 expression and the prognosis of glioma patients. However, the mechanism of GBE1 in Parkinson’s disease remains unclear. We speculate that GBE1 may also affect the glucose metabolism pattern of substantia nigra cells in a similar way to its impact on glioma cells, which could then lead to mitochondrial dysfunction and increased oxidative stress in these cells, and thereby contribute to Parkinson’s disease. However, this is only a speculation and requires further research.

Engrailed 1 (EN1) is a homeodomain-containing transcription factor that plays essential and widespread roles in the embryonic development of various tissues, including the cerebellum, midbrain, skeleton, and limbs (Loomis et al., 1996). EN1 has been shown to possess oncogenic properties in glioma, regulating cancer cell proliferation and growth through modulation of the Hedgehog signaling pathway (Chang et al., 2022). Previous studies have found that EN1 may become a common target for immunotherapy of PD and glioblastoma (Zhao et al., 2023). Another study has indicated that EN1 is involved in regulating the maturation and survival of dopaminergic neurons, potentially serving as a genetic risk factor for PD (Haubenberger et al., 2011).

The 19-gene signature is a predictive model based on gene expression data, which is used to evaluate the risk of tumor prognosis for individuals. This model enables clinicians to more accurately and effectively assess the survival status of patients. We found that patients in the low-risk group have a longer OS compared to the high-risk group. The model’s predictive performance was validated through external and internal dataset validation, confirming its independence from other clinical characteristics. As a quantitative tool, the DCA curve can assist in verifying whether the gene signature model can benefit patients by comparing the differences between actual risks and model-predicted risks to assess the value of the model. By plotting and analyzing the DCA curve, it can reveal the dynamic relationship between the prognostic model and other traditional clinical variables in terms of net benefit, thereby helping to more accurately estimate the cancer risk for individuals. Our DCA curves demonstrate that the application of our gene signature can benefit patients with gliomas, and it outperforms traditional clinical variables. Furthermore, we observed differences in the TME, levels of immune cell infiltration, immune gene expression, and drug resistance analysis across various risk groups. These differences could have significant implications for the clinical treatment of patients with glioma.

Transcriptome-based tumor prognostic models have certain advantages in predicting the prognosis of cancer patients. They can reveal changes in gene expression during tumorigenesis and development by analyzing the transcriptome data of tumor cells, thereby predicting the prognosis of patients. However, these models also have some limitations, mainly including the following aspects: First, the limitations of the quality and quantity of transcriptome sequencing data. In practical operations, errors may occur during sample acquisition, processing, and sequencing, as well as due to factors such as the heterogeneity of tumor tissues, which may affect the quality of transcriptome data. Second, the limitations and biases of transcriptome sequencing technology itself. Issues such as the comparability of data between different sequencing platforms and technologies, accurate measurement of gene expression levels, and detection of low-abundance genes may all affect the predictive performance of prognostic models. Third, the complexity of tumors and individual differences. Tumor is a complex disease involving interactions among multiple genes and pathways during its occurrence and development. Even if prognostic models based on transcriptome data can reveal the relationship between some gene expression changes and prognosis, they may not fully cover all the complexity and individual differences of the tumor. This may lead to the inability of the model to accurately predict the prognosis of patients in some cases. In addition, we did not conduct *in vivo* and *in vitro* experimental validations. In the future research, priority should be given to conducting *in vivo* and *in vitro* experiments to validate the mechanism of each gene within the gene signature in the occurrence and development of glioma and PD.

## 5 Conclusion

In summary, this study has identified a 19-gene signature composed of Parkinson-Glioma feature genes that holds prognostic value for glioma patients. Our research has proposed a predictive model and identified biomarkers for glioma patients, laying the groundwork for further mechanistic and therapeutic investigations.

## Data availability statement

Publicly available datasets were analyzed in this study. This data can be found at: https://portal.gdc.cancer.gov, TCGA, TCGA-Glioma; http://www.cgga.org.cn, CGGA, CGGA-325/CGGA-693; https://www.ncbi.nlm.nih.gov/geo/, GEO, GSE16011, GSE7621, GSE20141, and GSE49036; and www.genome.gov/GTEx, GTEx.

## Ethics statement

The studies involving humans were approved by the Ethics Committee of Qilu Hospital, Shandong University (Ethics ID: KYLL-2022(ZM)-073). The studies were conducted in accordance with the local legislation and institutional requirements. The participants provided their written informed consent to participate in this study.

## Author contributions

HZ: Formal analysis, Investigation, Software, Validation, Writing – original draft. JW: Conceptualization, Funding acquisition, Writing – review & editing. NS: Methodology, Resources, Validation, Writing – review & editing. NY: Investigation, Project administration, Supervision, Writing – review & editing. XW: Conceptualization, Funding acquisition, Supervision, Writing – review & editing. CL: Conceptualization, Funding acquisition, Supervision, Writing – review & editing.

## Footnotes

^1^ https://www.ncbi.nlm.nih.gov/geo/

## Supplementary material

SUPPLEMENTARY TABLE 1 112 differentially expressed genes in Parkinson's disease.

SUPPLEMENTARY TABLE 2 30 PGFGs discovered by eight machine learning algorithms.

SUPPLEMENTARY TABLE 3 19 prognostic PGFGs obtained through Lasso–Cox regression analysis and their coefficients.

SUPPLEMENTARY TABLE 4 Risk scores of patients in CGGA325.

SUPPLEMENTARY TABLE 5 Risk scores of patients in CGGA693.

SUPPLEMENTARY TABLE 6 Risk scores of patients in GSE16011.

SUPPLEMENTARY TABLE 7 Risk scores of patients in TCGA-Glioma.

SUPPLEMENTARY FIGURE 1 The workflow diagram of this study.

SUPPLEMENTARY FIGURE 2 Performance verification of different diagnostic models based on eight algorithms.
