# Supplementary figures and images for: Identification and validation of a novel Parkinson-Glioma feature gene signature in glioma and Parkinson’s disease
Source: Front Aging Neurosci. 2024 May 30;16:1352681. doi: 10.3389/fnagi.2024.1352681 (PMC11170708; doi:10.3389/fnagi.2024.1352681)

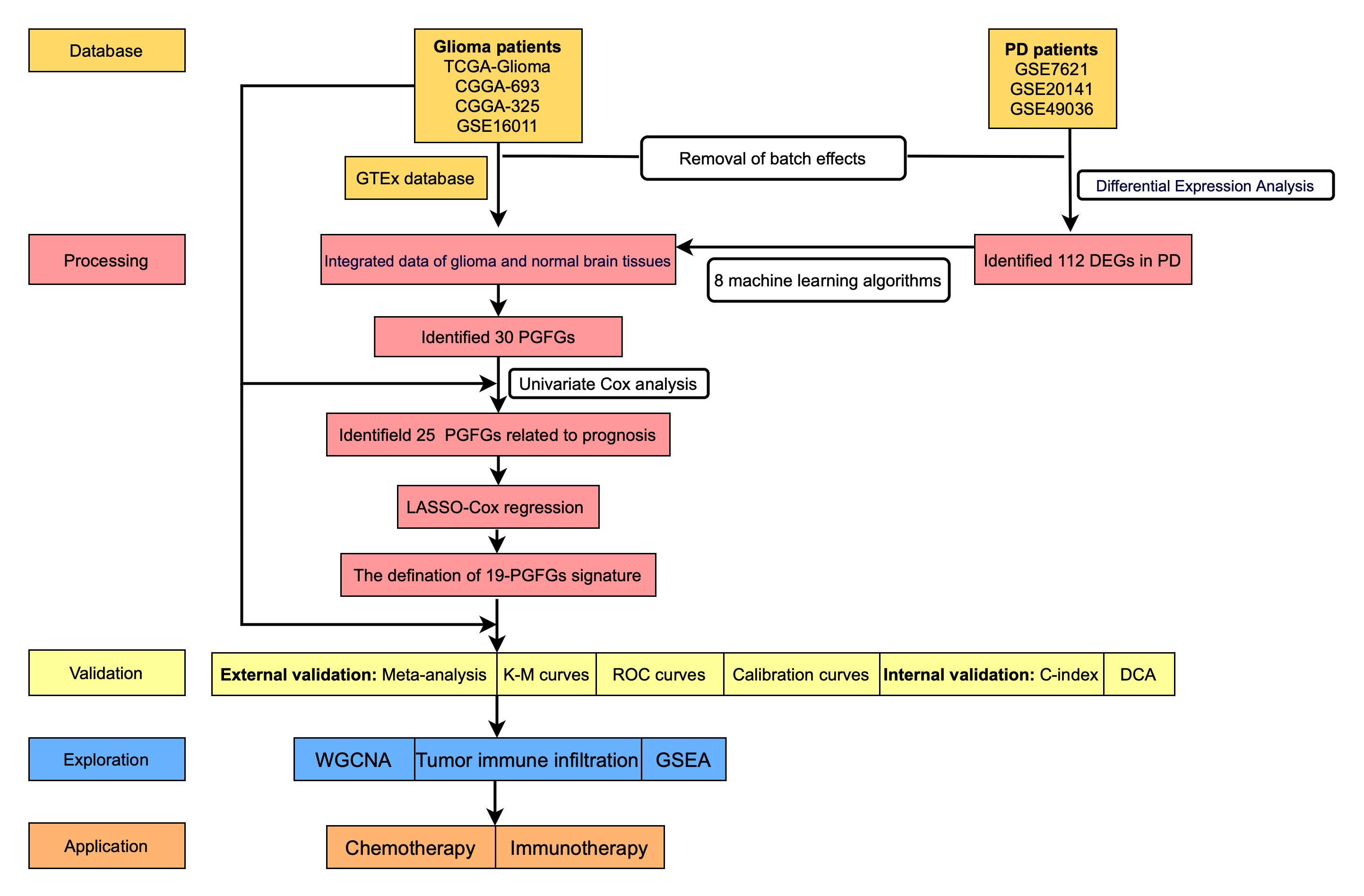

Supplement: SUPPLEMENTARY FIGURE 1 — The workflow diagram of this study. [file Image_1.PNG]

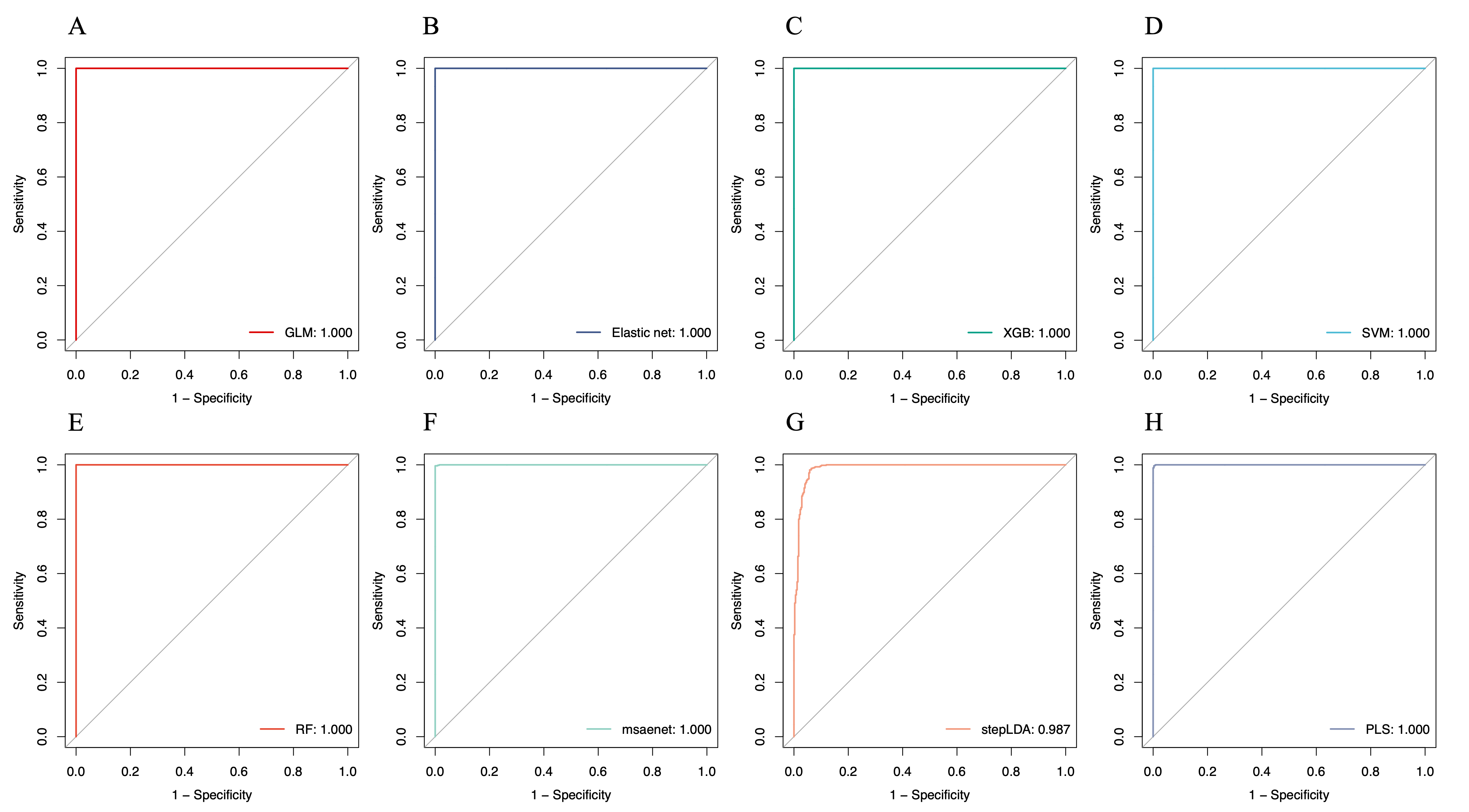

Supplement: SUPPLEMENTARY FIGURE 2 — Performance verification of different diagnostic models based on eight algorithms. [file Image_2.PNG]
